# “He Who Has the Spirits Must Work a Lot”: A Psycho‐Anthropological Account of Spirit Possession in the Dominican Republic

**DOI:** 10.1111/etho.12216

**Published:** 2018-10-29

**Authors:** Etzel Cardeña, Yvonne Schaffler

## Abstract

In this paper we present a multidisciplinary, developmental analysis of a Dominican Republic *Vodou servidor* (“Marcos”), from childhood to early adulthood, integrating ethnographic observation, field documentation, and anthropological analysis, with relevant constructs from developmental, personality, and clinical psychology. Marcos transitioned from a child with many problems to a young adult who has learnt how to control and adapt dissociative manifestations into a professional role as a Vodou priest. Most empirical studies on spirit possession (SP) have rarely taken a longitudinal, multidisciplinary approach, which may better account for such a complex phenomenon. Adopting such an approach helps make sense of both the continuity and changes in Marcos, as well as of his community's shifting attitudes towards him. We describe how specific psychological and cultural conditions help explain the change from originally dysfunctional expressions of SP to personally and socially beneficial ones. [anomalous experience, dissociation, gender, spirit possession, Vodou]

## Resumen

En este artículo presentamos un análisis multidisciplinario del desarrollo de un servidor del Vudú en la República Dominicana (“Marcos”), desde la niñez hasta la adultez temprana, integrando observación etnográfica, documentación de campo y análisis antropológico, con constructos relevantes de psicologías del desarrollo, personalidad, y clínica. Marcos se transformó de un niño con muchos problemas a un adulto joven que ha aprendido a controlar y adaptar las manifestaciones disociativas dentro de su rol profesional como sacerdote Vudú. Estudios empíricos sobre la posesión de espíritus (PE) han adoptado un enfoque longitudinal y multidisciplinario, que puede ayudar a entender mejor un fenómeno tan complejo. Tal enfoque ayuda a comprender tanto la continuidad como los cambios en Marcos, así como la transformación de las actitudes de su comunidad hacia él. Describimos cómo condiciones psicológicas y culturales específicas ayudan a explicar el cambio de manifestaciones originalmente disfuncionales de la PE a que proporcionan beneficios tanto personales como sociales.

## Zusammenfassung

In dieser Studie präsentieren wir eine multidisziplinäre Analyse der Entwicklung eines *Vodou*‐*servidors* („Marcos”) aus der Dominikanischen Republik, von seiner Kindheit bis ins frühe Erwachsenenalter, die ethnographische Beobachtung, Feldaufzeichnungen und anthropologische Analyse mit relevanten Konstrukten aus der Entwicklungs‐, Persönlichkeits‐ und klinischen Psychologie verbindet. Marcos entwickelte sich von einem Kind mit zahlreichen Problemen zu einem jungen Erwachsenen, der gelernt hat, seine dissoziativen Manifestationen so zu kanalisieren, dass er die Rolle eines professionellen Vodou‐Priesters ausüben kann. In empirischen Studien zu Besessenheit (B) wurde bislang nur selten ein multidisziplinärer, längsschnittlicher Ansatz verwendet, obwohl ein solcher dem komplexen Phänomen möglicherweise eher gerecht wird. Es lassen sich damit sowohl Kontinuitäten und Veränderungen in Marcos Selbst verstehen, als auch die wechselnden Haltungen seines sozialen Umfeldes ihm gegenüber. Wir beschreiben, wie spezifische psychologische und kulturelle Voraussetzungen dazu beitragen, dass ursprünglich dysfunktionale Manifestationen von Besessenheit zu solchen werden, die in persönlicher wie in sozialer Hinsicht nützlich sind.

The scholarly perspectives on spirit possession (SP) have been almost as multifaceted as the myriad of spirits who substitute the regular identities of human beings, whether welcome or unbidden. In the West, they go a long way back (Oesterreich 1921/1974) and include Plato's philosophical discussion of the *manias* in the *Phaedrus* dialogue, the centuries‐long Christian attempts to discern the nature of possessing entities (Sluhovsky 2011), and more recent discussions within anthropology (Bourguignon 1965; Boddy 1994), psychology (Mischel and Mischel 1947), neurocognition (Peres et al. 2012), dramaturgy (Métraux 1955), and parapsychology (Gauld 1984). However, most empirical studies have been cross‐sectional, evaluating one point of time, and have rarely adopted an interdisciplinary approach. In this article, we present the history of a *Vodou* devotee from childhood to young adulthood integrating anthropological and psychological perspectives.

The lack of longitudinal studies in SP studies hides important changes that may take place during the life of the experient. Seligman's work (2005; 2010) is a recent exception in presenting how initial distress and suffering manifested dissociatively may be transformed through the reinterpretation and reconstruction of a new, meaningful self within the context of *Candomblé*. Among Dominican Republic SP experients (*servidores*), general distress is also common, as well as a tendency to have episodes of psychological (e.g., amnesia) and somatoform (e.g., functional blindness and fainting) dissociation (Schaffler et al. 2016), phenomena that correlate among themselves and with a history of trauma (Brown et al. 2007); all three were more prevalent among individuals who experience SP than those who do not (Van Duijl et al. 2010). The term “dissociation” refers to experienced loss of information, memory, or control of psychological and somatic processes ordinarily available to conscious awareness, self‐attribution, or control (Cardeña and Carlson 2011); its manifestations can be functional or dysfunctional (Cardeña 1997).

Chapin (2008) presented an account of a 58‐year‐old Sri Lankan Kali priestess with a life fraught with serious difficulties, who transitioned from uncontrolled dissociative episodes to manifestations within a ritual setting including firewalking and blood sacrifices, to more sedate practices. Both Seligman and Chapin present longitudinal analyses emphasizing teenage and adult crises, but have little to say about earlier stages, which are crucial to understanding the development of the self. Furthermore, whether a manifestation is functional or not may change across time. This fluctuating status is exemplified by an SP Umbanda devotee who might have met criteria for DID (Dissociative Identity Disorder; a disorder characterized by dysfunctional discontinuities in the sense of self, accompanied by amnesia and other symptoms; Cardeña 2017) during her early life, but who no longer fulfilled them when she could control her possession states later on and found them meaningful (Delmonte et al. 2016).

Not all SP experients have preceding afflictions (e.g., Cardeña 1991; Davis 2007; Schaffler 2012), and only a few children and adolescents with psychological or medical problems will manifest later SP, so affliction must interact with other factors, such as a propensity to dissociate and have other unusual experiences, and “a culturally sanctioned medium for articulating … dysphoria” (Boddy 1988, 13). SP provides this medium in some cultures, in which it may be more or less functional. A developmental approach can enhance our understanding of which life contingencies and personality traits predispose an individual to dissociate more, and the reaction of the social environment partly determines how a particular manifestation will be valued. Developmental psychologist Jerome Kagan (2008) described how different historical times present varying adaptive challenges, with traits deemed positive at one point (e.g., pronounced guilt, during Puritanical times) and not valued at another (as compared with the diminished import of internal guilt in Western societies). Even within the same historical period and for the same individual, research on temperament (such as behavioral reactivity, consistent throughout the lifespan) shows that rather than assuming that a child's temperamental style is necessarily problematic, caretakers may value it positively if it “fits” their own personality characteristics, and their evaluation may change valence across time (Thomas and Chess 1984). For instance, parents who might have thought that their infant was too introverted as a small child may later value their “sensitive” teen.

With respect to SP, a good “fit” between its characteristics and what the culture prizes can help possession‐prone individuals to integrate the dissociative experiences in their lives so that they feel balanced and thrive (Delmonte et al. 2016), and possession‐based religions often have a positive effect on their members (e.g., Boddy 1988). Birman (2005) and Hayes (2006) argue, however, that even within a ritual context some individuals have limited control over the spirits that possess them, whose behavior, although expected, may go against traditionally accepted values. Although the transgression and excesses that distinguish some spirits may help express usually prohibited aspects of the self (Boddy 1988), they can also be problematic. This is the case when they provoke quarrels and misunderstandings by revealing embarrassing information, air personal disputes, or deliver pointed insults (Bourguignon 1976, 33; Hayes 2006, 158). Some dimensions of SP such as controllability, personal and social meaningfulness, and subservience to ritual principles make it more likely that it will not only attract social approbation but also help the individual integrate diverse aspects of the self. An integration of anthropological and psychological concepts can validate each other and provide a more complete analysis of SP. We provide a brief description of Vodou in the Dominican Republic, give an overview of the life of a current devotee, Marcos (not his real name), and then discuss psychological and anthropological models that help explain his developmental path.

## Vodou in the Dominican Republic

In the Dominican Republic, vodouists self‐identify as Catholics, although only a fraction of Catholics hold a belief in Vodou, in which SP by entities commonly but not exclusively associated with Catholic saints is a central feature. SP often takes place during religious celebrations after invocation through music, dance, and bell ringing, but the possessing entities may also manifest outside the ritual setting. Dominican Vodou is similar to and influenced by Haitian Vodou, although far less widespread (Davis 2007). How it is viewed depends on the socioeconomic and cultural divisions between the professional class and the largely disenfranchised communities of informal workers in rural or suburban areas. Particularly Pentecostal congregations and other independent Christian churches are vehemently opposed to Vodou and consider its spirits (*misterios*) to be demons (Schaffler 2012; 2013).

A lack of material resources and infrastructure has rendered Vodou a de facto health system for many in the Dominican Republic. Although biomedicine is well established and going to the doctor is one of the first options when Dominicans feel unwell, public hospitals in rural areas are often badly equipped, and many individuals with psychological and related symptoms might contact not only biomedical doctors but also herbalists and religious healers, some of them vodouists (Schaffler 2009; 2017). In a previous study, we found that 34% of possessed individuals (*n* = 47) had sought medical advice for spontaneous dissociative phenomena. After not receiving the help they were expecting from very basic medical examinations, they interpreted their symptoms as signs of SP (Schaffler et al. 2016). For instance, a young woman suffering from dissociative episodes with violent features reported that they were ultimately attributed to SP by a distinct class of *misterios* (spirits of uncivilized indigenous people). She found relief after being initiated into Vodou and learning how to control her “wild” possessions (Schaffler 2017).

## The Story of Marcos

This account is mostly based on two narrative interviews conducted and audio‐recorded by the second author (YS) with Marcos, 20 years old at the time of the first interview. The purpose of narrative interviews is to acquire accounts of subjective experiences that can be later analyzed (Anderson & Kirkpatrick 2016). The first interview took place during April 2012 and dealt with Marcos's spiritual development and SP. The second was biographical and was conducted in January 2013. YS approached Marcos through the help of a well‐known long‐term practitioner of Vodou who introduced her to several of his peers around the towns of Baní and San Cristóbal. According to that practitioner, Marcos's former suffering was remarkable, followed by an impressive “spiritual development” through which he has turned into a capable priest. He interprets initially unwilled possession, as in the case of Marcos, as a sign of the veracity of a spiritual call. More information on Marcos's case was gathered during informal conversations and subsequent entries in a field diary, through filming a public vodouist celebration organized by him in May 2012, and by administering him a questionnaire on the phenomenology and triggers of possession trance in 2013 (Schaffler et al. 2016). The story of Marcos is part of a project comprising narrative interviews focusing on the life and spiritual development of 19 participants. The data include interviews with questions about their religious practices, videographic recordings in ritual contexts, and participant observation including informal interaction with participants. Other data from the project are referenced as needed. The research was approved by the Ethics commission of the Medical University of Vienna (141/2011). Participants signed an informed consent form and got written and verbal information about the aims and procedures of the study.

### Early Childhood

Marcos's early life was anything but easy. His mother, a teenager at the time he was born, gave him up to his maternal grandmother before he could walk. Although it is not that uncommon for young mothers to leave their children with grandparents while they seek work someplace else, Marcos reiterated that he never received support from his parents, who “threw him away,” and only showed interest in him after his spiritual vocation was recognized. He also stated that he survived thanks to “the help of God and the spirits.” He grew up in an impoverished rural area formerly dedicated as a sugar plantation (a *batey*) in the Dominican East, several driving hours away from his birthplace in the South. He states that the only contact with his mother was an occasional call. His father married another woman and had children with her. In his rare visits, he beat Marcos up and threatened to kill him because of his being a “maricón” (“faggot”). As a child, Marcos preferred to play with girls and liked to help in the house with cooking and cleaning. Although Marcos's grandmother accepted this behavior and defended him against his father, she too used to beat him “a lot” when he was little in an attempt to vanquish the demons causing his frequent “insolent behavior.” When the boy felt bad and needed her attention, she assumed that he was simulating.

### Middle Childhood

Marcos was eight years old when he experienced what he today classifies as his first episode of possession: during school he suddenly lost consciousness and fell to the floor. From then on, he would experience loss of control over his body several times a day, often feeling like he was being dragged or thrown around. This used to happen also in the street, at home with family and friends, and when he was taken to religious celebrations. He also occasionally became temporarily blind, and, because he found eating difficult, remained very thin.

During school, Marcos was unable to focus and failed to even learn how to read and write. He pointed out in 2013 that he continued to have problems learning new material because he “forgets everything.” On top of that, his classmates beat and mocked him although his teacher, whom he helped clean up and decorate the classroom, did her best to defend him. Several doctors evaluated him for blood pressure and diabetes but not, as far as he mentioned, for seizure disorder, and a psychologist whom they consulted could not provide any diagnosis. Fortunately, a maternal aunt who lived in another town and was very interested in Vodou became Marcos's protector and guide and interpreted his playing with saint figures and matches. To her, these were no childhood games but enactments of spiritual celebrations (*fiestas de palo*) in honor of the spirits of Vodou. Together, they tinkered a hand bell from the cap of a beer bottle and a nail. Within the vodouist framework, a hand bell is a powerful tool to call on the spirits (*misterios*). That aunt also encouraged the fantasy life of her children and Marcos by making rag puppets for the children to play with. When Marcos started enacting vodouist rituals while playing with other children, around seven to eight years of age, the grandmother, a very faithful Catholic, was not supportive of his interest (as in the case of Delmonte et al. 2016), but she soon changed her mind. She informed his father about it and even used numbers that Marcos predicted would be chosen at the lotto. His “gift” was discussed in the neighborhood, and people came to see him so that he would predict their future.

When Marcos was in fifth grade, he felt even worse because “the spirits” would cause him to lose control of his body and not let him sleep or eat. Eventually his schoolteacher, who likewise supported a spiritual explanation for his suffering, approached his grandmother requesting her to take Marcos out of school. Convinced that his symptoms resulted from incomplete possession and spiritual punishment because the spirits could not fully enter and make use of his body, the grandmother took him to a healer to help him develop his “light.” In Dominican Vodou, formal training is minimal (Davis, 2007, 79). Healers pass their knowledge during private healing sessions while being in a state of possession (so that the knowledge is delivered “directly” by the spirits) and through informal conversations that take place outside the ritual context. Marcos found some relief after spiritual cleansing baths in ritual places and was taught techniques to “work with” (i.e., control) the spirits, such as appeasing them with offerings, going on pilgrimages, or binding them with a string that he applied to his leg. At this point, his grandmother had become his ally and accompanied him to pilgrimage sites and vodouist celebrations he had to attend so as to “develop his spirits.” On such occasions novices meet more experienced mediums and dance together while in possession. They are taught interactively and are directed to move according to the guidelines of Vodou. The “teaching spirit” may communicate, for example, details of a ritual salutation unknown to the possessed novice. In other situations of knowledge transmission, the “teacher” may not be possessed but serve as a ritual assistant (plaza). In these circumstances, the experienced medium handles ritual paraphernalia (e.g., a hand bell used for calling the spirit) and applies and manages prayer, perfume, food, and drinks to regulate if and how the novice becomes possessed (Schaffler & Brabec de Mori 2016).

### Adolescence

At age 13, the process of his initiation, which consisted of several rituals called “refreshings of the head,” a self‐organized pilgrimage and a public *fiesta de palos* was completed. He had learned how to manifest the spirits in a “firm” manner, meaning that he would not fall anymore when the force (spirit) entered or left him. However, he still had difficulties in controlling his movements when the spirits entered his body. While hosting the celebration that marked his inauguration as a fully initiated *servidor de misterios*, “all 21 spirits” mounted him subsequently “from eight in the morning until eleven at night,” leaving him extremely exhausted. Nevertheless, he felt ready to “work” the spirits in a professional setting. He read the cards to predict people's future, and healed and counseled them while possessed, in a tiny wooden shack near his grandmother's house. At that time, the contact with clients made him anxious.

Throughout his spiritual career, his ability to control the spirits depended on his willingness to take care of them, which included cleaning the altar, preparing the right offerings, organizing celebrations, and visiting sacred places. As is common among vodouists, the spirits told him through dreams, spontaneous inspirations, and signs in the environment how these activities should be done. Among his patron spirits were Saint Michael (or Belié Belcan), a protecting and moral authoritative male, and two females (*metresas*), the determined Santa Marta, and the coquettish Anaísa. If he failed to attend to their precise needs, the spirits would punish him by “pushing him over” so that he would fall or give him a stomachache. He stated: “He who has this (the spirits) must work a lot. (He experiences) much turbulence because things do not run smoothly, things work only if God and they give their permission. After I take care of them, they give me a little peace, but if I don't, I keep feeling sick in my stomach. If I refuse to attend to them, I cannot eat and do other things… I have to work really hard.” He cleaned the altar and carried out related activities to thwart involuntary possessions. Besides the problems Marcos attributed to the spirits, during puberty his attraction to men caused him so much shame that he barely spoke or left the house.

At age 14, he lost consciousness at a pilgrimage site and was unresponsive for hours. According to his interpretation, he had failed to visit the pilgrimage site at the right time. He assumed that the spirits Anaísa and Saint Michael were jealous of each other and fought for supremacy in his head (*cruce de misterios*). Another basic medical check‐up failed to offer a medical explanation. Around that time, he followed a friend and visited a Pentecostal worship service in an attempt to alleviate his ongoing suffering (he reported in a questionnaire that he had had violent possessions before initiation several times a week), but he felt sick and cold before entering the church. During the service, he lost consciousness and fell to the floor. Although the members of the church encircled him to perform their deliverance ceremony, he felt that the spirits were beating him so he freed himself from the group and never went back.

In the same year, Marcos's beloved maternal grandmother “died in his arms” from heart disease. After grieving his loss, he returned to the South and moved in with his paternal grandmother who lived near his parents in a shantytown. Things started to get gradually better. Feeling less depressed, he left the house and explored his new neighborhood. He started meeting other *servidores* and felt that he “gained in spiritual force” as his grandmother had passed on her “light” to him. Although she had initially rejected anything “spiritual” besides Catholicism, she was, according to Marcos, a “seer” (*vidente*). He wrote that before his initiation he suffered “very much,” but his affliction became “moderate” during the initiation and further decreased after it. He also stated that at the time of the second interview, “violent” possessions tended to happen only in private and “moderate” suffering. Some of his experiences during SP included becoming insensitive to pain, not sensing the body or parts of it, seeing things as different than usual, and being only able to whisper or not speak at all.

### Young Adulthood

After things had become better for Marcos around the age of 15, he identified himself as homosexual and had his first relationship with a man when he was 18 years old. He says: “This was a good relationship (although) I was ashamed because it was my first time. I felt good, and he felt good. But he was married and now has a wife and two kids in Boston… Once he called me and told me that he wanted to get involved with me again … I have had a lot of lovers but I don't like it like this. (I prefer) one alone.” Around that time, he also bought a small piece of land with the money his maternal grandmother left him. His maternal grandfather frequently travelled to see him and helped him build a wooden shack. Being the owner of his own house, Marcos now worked as a servant of the spirits (*servidor, brujo*) on a more professional level. By the time he was interviewed, he was using business cards. The services he provides include diagnosing the spiritual root of his client's problems while possessed, treating them by administering cleansing baths, magical medicines, and talismans, and harnessing the power of the spirits. He states: “Earlier on, everything was a struggle, a mockery. The spirits mounted me in the streets and during funerals, and people thought that I was joking… But when I settled in my own house, everyone started coming to me… Now they respect me…. People support me wherever I go. It looks like God arrived.” With the help of a client, Marcos soon extended his shack and built an additional one to host his growing altar. Another client thanked him by giving him an additional piece of land.

At the time of the first interview (2012), Marcos still occasionally suffered from headaches, back pain, vertigo, sickness, a queasy stomach, paresthesias, and an inability to feel or move his limbs. He also exhibited tics like hand clapping and uncontrolled eye blinking and spoke with a stammer, which has been related to social anxiety (Iverach and Rapee 2014), and dissociative symptomatology (Cardeña and Gleaves 2007). During the second interview (2013), he seemed more relaxed, spoke more coherently, and did not have tics. Still, he complained of sleeplessness and other symptoms. In the questionnaire, he reported that during rituals the spirits would still mount him several times a week without being invoked, which he said he did not mind. However, in situations he felt uncomfortable, the spirits would also drag him around and throw him onto the floor, which would cause him “considerable” suffering, particularly as this also happened when he was with family and friends. He said that he regularly “feels the presence of the spirits” like a positive force that surrounds him. Sometimes, however, they would feel like a burden on his chest.

Despite identifying as homosexual, at the age of 20 Marcos initiated a relationship with a woman who, after she had occasionally seduced him when he was drunk, became pregnant. Because he did not want her to abort the baby, he invited her to live with him, but he was neither sure that he was really the father of the unborn child nor that he enjoyed living with the woman: “I am with her but I have my hidden boyfriend. In fact, living and having relations with her disgust me.” Also, his patron spirit Anaísa had signaled to him that being with a woman was a poor decision, and people asked him why a gay man would live with a woman. Marcos decided to “rid himself from that cross,” expressing his confidence that God and the spirits would help him.

Another difficulty in his adult life was that having too many clients stressed him. Mounting many spirits consecutively during counseling sessions or *fiestas* left him exhausted and with a “full head.” The exposure to “heat” (arousal), particularly when mounting the vigorous spirit of Saint Michael, decreases his ability to control the spirits, and the excessive consumption of cigars and liquor, both usually demanded by the spirits possessing him, worsens his health. All this is problematic because “working” the spirits in a way that their messages come out clearly requires high concentration and a “clean mind.” To reduce the risk of being “abused by the spirits,” Marcos limits the number of clients he is willing to serve to five a day. When visiting spiritual celebrations hosted by peers, he “binds” the spirits with an amulet on his ankle to prevent them from rising “up to his head” as long as he wears it. Moreover, since his doctor advised him to drink less to ease his stomach problems, he has come to an agreement with his spirits not to demand liquor but wine during the ritual.

At the time of the second interview, Marcos had not only rebuilt his house but also connected a small chapel to it in which he placed his altar and hosted well‐attended public spiritual celebrations. He performs his ritual duties systematically and with grace. During his possessed interactions, when saluting, blessing, or counseling his clients, he is aware of them and stirs positive emotions in them. Although Marcos does not have professional or technical training, he earns his living and is recognized as the leader of his spiritual center.

## Analysis of the Case of Marcos

### The Selves of Marcos: A Psychological Perspective

The first major compendium of psychology was William James's *The Principles of Psychology* (1890), in which he listed different constituents of the self (material, social, spiritual, and pure Ego). He saw them as processes in which, for instance, different feelings and emotions give rise to self‐feelings and actions. He also described dissociative or alternating identities, including those found in “mediumship” and “possession” without pathologizing them. James was aware of the historical and cultural relativity of experience and has been called a pioneer of constructionism (Bruner 2004). Closer to our time, Neisser (1988) provided another classification of aspects of the self: ecological/sensorimotor (the bodily self within the surrounding environment), interpersonal (the self in relation to others), extended (the self across time, with reference to ancestors or looking forward to the future), private (mental interiority not shareable with anyone else), and conceptual (the person's various attributions about him/herself). SP affects most of these aspects of the self: the *servidor* experiences that she or he loses control of his or her body (i.e., ecological/sensorimotor aspect), and that it is an “other,” a *misterio*, with a different personal history and characteristics (i.e., interpersonal, extended, and conceptual aspects) who interacts with others; the *servidor* typically states not being able to remember what transpired during the SP (private aspect). This analysis helps differentiate SP from “trance” experiences in which the person experiences losing control but not becoming a different self with personal history and characteristics (Cardeña et al. 2009). It is also worth noting that SP is intrinsically an embodied alteration of consciousness (Cardeña 1989; Seligman 2010; Halloy 2012; 2013; Pierini 2016a; 2016b; Schmidt 2016), often elicited by robust bodily interactions (Rouget 1980) rather than a mostly imaginal state (Cardeña and Krippner 2018).

Although (sub)cultures differ in their views of conceptual aspects of the self (e.g., Markus and Kitayama 1991; Kirmayer 2007), even within more collectivistic cultures like the Dominican Republic, and even among Vodou followers, only a minority exhibit SP as Marcos does, so a discussion of individual differences and of the contingencies of the development of the self is relevant. The development of a coherent self (or, rather, of different aspects of it) is not innate but an achievement that goes through stages. Stern (1985) distinguished developing aspects of the self, with the initial two referring to an integration of sensory stimuli and distinguishing self from others. Putnam (1997) emphasized that an infant must organize and learn to regulate different experiential, emotional, and behavioral states (e.g., learning how to regulate crying outbursts), and that achieving self‐regulation and a coherent sense of self can be hindered by trauma, abuse, or neglect, particularly by the primary caretakers.

The relationship with the main caretaker(s), on whom the child depends completely, is the most important aspect in the early development of the self (barring severe innate or environmental deficiencies). Caretaker(s) experienced as providing security and a safe emotional haven can model to the child a sense of inner coherence through their interactions with him or her, and the child can develop organized models of the self and the surrounding environment. Contrariwise, caretaker(s) who are consistently punitive, neglectful, or unpredictable may elicit models of self that reinforce experiential fractures and a lack of bodily and experiential control (Lyons‐Ruth et al. 2006). Cultures differ in their child‐rearing practices, but there is substantial support from cross‐cultural research for a universality of attachment behaviors and a link between sensitive caretaking and a secure sense of attachment to the caretaker(s) (Mesman, Van Ijzendoorn, and Sagi‐Schwartz 2016). After childhood, self‐representations become increasingly more fraught in additional interpersonal distinctions and comparisons with peers and with the roles that one is expected to adopt at that time (Harter 1999).

With this psychological framework in mind, we can try to make sense of the early life of Marcos, starting with the abandonment by his parents. Although he was raised by his maternal grandmother (we will come back to her), the parental abandonment likely disrupted the development of a basic sense of attachment (or basic trust, in Erikson's model, 1950) and a concomitant sense of an integrated body with emotional episodes becoming increasingly self‐regulated (instead of being extreme and uncontrolled), and states of consciousness (e.g., dreaming and awake mentations) becoming differentiated (Putnam 1997). The caretakers have a primary role in this development by soothing, modeling self‐regulation, and helping interpret and organize the different aspects of the infant (Lyons‐Ruth et al. 2006). The abandonment (which Marcos expresses as being “thrown away”) by his parents may thus partly explain his early symptoms: syncopes, episodes of derealization, problems with eating, and so on. In other words, the aspects of the self described by Neisser and Stern are likely to have been more compromised than those of a child raised from the beginning by consistently loving caretakers.

We do not know whether Marcos was evaluated for seizure disorder, but the complex of symptoms, including medically unexplained fainting spells and experiencing the environment as not quite real, also matches those of nonorganic seizures, which often have some form of maltreatment and/or serious adverse events in childhood as a causal factor (Campo and Fritsch 1994; Litwin and Cardeña 2001; Pick, Mellers, and Goldstein 2017) and suggest dysregulation of different psychophysiological systems (cf. Griffin, Resick, and Mechanic 1997; Putnam 1997). The likely development of an insecure type of attachment given the physical and emotional abandonment by his parents and the threats by his father (and perhaps also the physical punishment by his grandmother) would predict his psychological and somatic dissociative reactions (Liotti 1999).

Nonetheless, we must also explain why Marcos has shown some resiliency, particularly in recent years, despite his early severe hindrances, although it is important to bear in mind that disturbed children may become healthy just with the passage of time (Kagan 2008). Marcos had to cope with the abandonment by his parents, but two other figures provided support. His maternal grandmother, although initially punishing him physically and condemning him for his religious choice, took over the role of a parental figure, providing material and emotional sustenance and even protecting his budding sexual identity from the assaults of his father and the condemnation by his peers. Furthermore, as is common in countries where psychological services are limited (Van Duijl, Kleijn, and de Jong 2014), traditional religious healing played an important role in how Marcos dealt with distress. At the beginning, his grandmother did not support his inclination to devote himself to the *misterios*, but at that point an aunt and a teacher supported that interest, and the cultural explanation of possession by the *misterio* spirits became a framework to explain his symptoms. Dependable social support, provided in this case by some members of Marcos's family and the community of *servidores*, is a predictor of resilience among those growing in disadvantaged social milieus (Werner 2005). Moreover, transmission of esoteric teachings by Vodou teachers, following meticulously ritual precepts, and observing how others regulate their *misterio* manifestations provided models of how to deal with initially uncontrollable psychophysical states (Halloy 2012).

### The Unusual Experiences of Marcos

Marcos is far from being the only child who has been physically and/or emotionally abandoned or abused by caretakers, yet only a minority of them exhibit marked dissociative phenomena. At this point, genetic personality predispositions provide a supplementary explanation. Even within his social milieu, Marcos's SP experiences are unusual, but this might be partly accounted for by his genetic disposition to dissociate and have transcendental experiences. Various studies (conducted almost exclusively in industrialized countries) have found substantial heritability for dissociation (reviewed in Dalenberg et al. 2012), as well as for interrelated constructs that predict the likelihood of having anomalous experiences, including responsiveness to hypnotic suggestions, absorption, thin mental boundaries, and self‐transcendence (reviewed in Cardeña and Terhune 2014). Heritability explains about 50% of the variance of these traits and interacts with developmental events. For instance, high hypnotizables who are also highly dissociative tend to experience less psychological control, have problems with memory, and report more exposure to traumatic/stressful events than those who are not dissociative (Terhune and Cardeña 2010).

Marcos's spiritual experiences are consistent with the personality trait of self‐transcendence (ST), referring to being intuitive, acquiescent, spiritual, and sensing an interconnectedness with an unperceived whole (Svrakic et al. 2002). Our reasons for focusing on ST are that: (1) it conceptually matches some of the experiences of Marcos, (2) it has shown validity in different parts of the world, (3) its relation to psychological health or pathology has been investigated, and (4) it correlates strongly with absorption, a construct that has been posited to mediate religious experience (Luhrmann, Nusbaum, and Thisted 2010), and with “thin mental boundaries” (Cardeña and Terhune 2014). The latter construct refers to not making sharp distinctions between psychological processes (e.g., states of consciousness) or senses of the self (e.g., gender identity) and is consistent with Marcos's propensity to transit among states of consciousness and have a nonstereotypical sexual identification. Dissociative and other unusual experiences are not necessarily dysfunctional and may allow individuals to demonstrate capacities not available to their regular selves such as creative automatic writing, imperviousness to pain, and even ostensible psychic phenomena (Cardeña 1997; Cardeña, Lynn, and Krippner 2017). Some of these experiences and capabilities are consistent with models of a permeable self. Along these lines, Strathern (1988) described a mode of personhood that proposes persons as relational and composed of divisible components and coined the term “dividual.” In the context of SP, dividual aspects of the self may account for the connections of a person with other social and even spiritual entities (Pype 2011). Connecting the perceptible human world with other experienced aspects is consistent with but goes beyond the characterization by Lester (2017, p. 9; see also James 1890) of the self as “epiphenomenal, arising from a buzzing, dynamic *network of internal relationships* rather than as any sort of identifiable, stable… state of being.”

Although self‐transcendence by itself has been tied to psychological dysfunction (Bayon et al. 1996), in conjunction with high self‐directedness (initiative) and sociability it predicts good psychological health (Cloninger 2003). This finding, based on group data, is consistent with Marcos's case. His initial fainting spells and other symptoms, besides his alienation from school, might have culminated in chronic dysfunction. Yet Marcos's account describes someone who was both supported by some in his social milieu and became self‐directed from an early age to learn more about how to become a *servidor*. From a relatively early age, Marcos was declared by others to have an important call, which he pursued, with the support of those around him with whom he interacted. Marcos gradually switched from his initial social isolation to expanding his social network, first as a spiritual novice and now as a spiritual leader. The change from afflicted boy to recognized spiritual expert has largely depended on Marcos's increasing sense of purpose and growing competence in Vodou knowledge and practice, and his and his surrounding milieu's acceptance of his identity as a *servidor*, in agreement with Erikson's (1950) scheme of identity development from late infancy through adulthood.

To be functional, there must be a good match between a person's identity and the acceptable surrounding cultural roles. A helpful concept to understand the change from Marcos's distress during his early years to increasing equilibrium is the genotype/environment “goodness of fit” proposed by Thomas and Chess (1984). They found in their longitudinal research that whether children are considered “easy” or “difficult” not only depends on their temperament but on how well it matches the characteristics prized by others. As in a Brazilian case described by Delmonte et al. (2016), Marcos's unusual early behaviors and experiences did not have a “good fit” with his grandmother (initially) or school peers, but they increasingly became reinterpreted and encouraged when his aunt and others saw them as a valuable spiritual call that should be heeded and channeled in a positive way. His aunt encouraged his imaginal life rather than condemning it, similarly to what happens among some children who develop high hypnotizability (Hilgard 1974), which might have also gradually decreased Marcos's anxiety and the occurrence of the more dysfunctional manifestations such as not eating and fainting at inopportune occasions.

### The Psychopathology of Marcos?

Contra the opinions of authors such as Spiro (1997), actual research with SP devotees shows that they are not as a rule psychotic nor do they necessarily exhibit more psychopathology than their referent groups, although some of them are dysfunctional even from an *emic* perspective (for reviews, see Boddy 1994; Cardeña et al. 2009). Thus, SP could be considered an *anomalous experience* (AE), an unusual or atypical experience that is not necessarily pathological (Cardeña, Lynn, and Krippner 2017). Some SP practitioners derive personal and social benefits from their SP, although others, often in what Lewis (1989) called *peripheral* forms of possession, exhibit violent and uncontrolled features that may provoke personal suffering and remain unintegrated with local ritual practices. Examples include *caballo lobo* in the Dominican Republic (Schaffler 2012, 2013), in which SP manifestations are destructive and contradict the conscious interests of the possessed (Hayes 2006; Schaffler 2017). Thus, SP should not be considered to be homogeneously beneficial or negative even within a specific culture, and Nichter makes a cogent case to treat *idioms of distress* as dynamic processes that may at different times provide “culturally and interpersonally effective ways of expressing and coping with distress” (2010, 405) and at others may be maladaptive, as seems to have been the case with Marcos.

Psychopathological classifications are *fuzzy* categories, and whether an experience is harmful or not depends on various factors such as controllability, absence of other symptoms, acceptance by the social milieu, potential for personal growth, and so on, with no single criterion determining what is/is not pathological (Moreira‐Almeida & Cardeña 2011). An important distinction with medical disorders is that whereas in them a determinable condition such as very high blood sugar levels can produce a number of symptoms (i.e., diabetes), in psychopathology diagnoses are based on correlations between diverse psychological experiences and behaviors (Borsboom 2017). Under what cultural circumstances and for which people these experiences are positive or not requires a consideration of multiple factors. Cardeña, Lynn, and Krippner (2017) propose that a genetic predisposition for AE may become dysfunctional depending on developmental vicissitudes (e.g., secure vs. insecure attachment histories); the absence of independent psychopathology (e.g., cognitive disorganization); flexible, effective coping mechanisms, and other personality factors (e.g., self‐initiative and sociability), all within the framework of the acceptable beliefs and practices of a culture.

In the case of Marcos, from an *etic* perspective his early manifestations of syncope, eating problems, and probable depression and anxiety might have justified a diagnosis of a functional neurological (DSM‐5; APA 2013) or dissociative (conversion) (ICD‐10; World Health Organization 2017) disorder, considering the suffering and dysfunction they engendered, provided that seizure and other medical disorders were ruled out. In the first interview, Marcos manifested considerable anxiety, and his manifestations were still often uncontrolled, but by the second interview his uncontrolled SP episodes were more organized according to ritual needs, he was considerably less anxious, and he had gained recognition and an economic foothold in his community as the head of his spiritual center. He has established his identity as a spiritual leader in the community who can support himself through his services. At this point, he would likely not fulfill the criteria for a psychological disorder. An unsolvable question, though, is whether a good therapeutic intervention early in his life, centered on his experiences of abandonment and his probably psychophysiological dysregulation, would have helped him, independently of his adopting Vodou practices or not.

### Marcos's Reinvention of the Self: An Anthropological Perspective

There are various cultural aspects relevant to Marcos's case. Let us start with the notion of a *spiritual call*. In Western industrial cultures, Marcos's affliction would have been typically attributed to a neurological or psychological disorder that should be treated. However, in the last few decades, alternatives to the medical model have proposed that experiences such as those of Marcos might be better considered as *exceptional experiences* (White 1997), or *spiritual emergencies* that should receive specialized support to become positive (Grof and Grof 1989), rather than being suppressed. These alternatives, however, have fostered little research within psychology and psychiatry (cf. Lukoff 2011), whereas the anthropological literature offers various observations in which an original medical or psychological crisis might culminate in a prized role as a shaman or SP expert (Halifax 1981). For instance, Stépanoff (2015) points out that among the Siberian Tuva people the *call* to become a shaman is violent and unwilled initially (as can happen with *servidores* in the Dominican Republic; Schaffler 2012; 2013), but the shaman later becomes an esteemed member in the community.

In the Dominican Republic, once a manifestation is identified as a *misterio* call rather than a psychological or medical disorder, potential *servidores* are provided a positive reinterpretation and specific techniques to help them bring it into increasing control, potentially turning a dysfunctional into a functional manifestation (Douglas 1966; Seligman 2005). Even in recognized severe psychopathology such as schizophrenia, reinterpretations of hallucinations can be beneficial (Romme and Escher 2012). The Vodou explanation for gaps in experience and behavior as the manifestation of *misterios* and the consequent prescriptions for how they can be handled ritually can be seen as a therapeutic as much as a spiritual strategy. Marcos's afflictions in the beginning were explained *emically* as attempts by the spirits to mount him that were unsuccessful because his body was not prepared to receive their force, which is why he had to undergo ritual initiation. After this process was completed at age 13, an alternative explanation for Marcos's ailments gained in importance. Now the spirits were angry at their horse and punished him for not completely fulfilling his various ritual duties, a condition locally referred to as *castigo* (punishment) (Schaffler 2017), which provides a potential explanation whenever a manifestation ran counter to positive expectations.

Vodou not only provided an explanation of Marcos's afflictions but had also a very important role as an explanation for his sexual identity and ensuing emotions, very much within general frameworks of symbolic healing (Dow 1986). SP allows for culturally accepted forms of otherness, with the potential for a resolution of ambiguities within the “ordinary” self (Boddy 1988; Kirmayer 1994). By his own account, Marcos's discovery of his gay sexual orientation was a source of great shame and social stress, particularly considering that he lives in a strongly heteronormative culture in which that orientation is stigmatized (Meyer 2003). A deviation from traditional gender roles can be severely punished, as exemplified by the threats of his father. Marcos was fortunate to have had the support of his grandmother and that of the Vodou ideology in which the diverse personalities of the spirits represent a range of gendered possibilities not offered by Roman Catholicism (McAlister 2000). As in *terreros* for Macumba groups in Brazil (Fry, 1985), in some urban or suburban Vodou centers in the Dominican Republic, the greater part of the male devotees is homo‐ or (although older practitioners who feel committed to Roman Catholic values frequently criticize this development, see Schaffler and Brabec de Mori 2016). Unlike in Boddy's example of possession (1988), in which the *zairan spirits* represent forces alien to those whom they possess, in Vodou a permanent link is assumed between devotees and their patron spirits, as their personalities tend to coincide (McCarthy Brown 1991, 112–13). If a youth has a male body but experiences a feminine identity, an explanation is that he is permeable to the influence of one or more female *misterios* (in particular Anaísa and Santa Marta). This may transform one's identification from being in a shameful group to that of being affiliated with a powerful female spirit. There are at least four ways in which the ideology and practice can help express and give meaning to male homosexuality: First, “being mounted” by the spirits is redolent of the prototypical heterosexual act in which the male often is talked about as mounting over and dominating the female, an association that Dominican *servidores* state themselves (Marcos mentioned taking the “receptive” role in his gay relationships). Second, being possessed by female spirits provides Marcos with accepted expressions of femininity within the Vodou pantheon, although by placing a large statue of the Archangel Michael or Belié Belcan in the most prominent position of his altar he also marks the masculine aspect of his spirituality, involving Michael's association with truth, justice, and “everything that is right.” Third, observing others “being mounted” by *misterios* of another gender gives Marcos experiential companionship, a frame of reference, and potential models for shared learning among practitioners of Vodou (Halloy 2012). And fourth, learning the signs of SP, for instance interpreting the ringing of a hand bell as a call to the spirits, provides a guiding framework to the experience. Furthermore, SP ceremonies can be just enjoyable, transgressive in sexual and other ways, and humorous, as the authors of this article have observed in different countries (see also Boddy 1988).

Finding an accepted way to reidentify himself as an expert on ritual services elected by the *misterios* rather than being a discarded sick being has Marcos a meaningful and profitable activity in the midst of various supporters, some, but not all, gays. Nonetheless, his conflictual intimate relationships, including his uncertain relationship with a woman, makes us suspect that he continues to struggle with what Erikson (1950) called the early adulthood crisis of intimacy versus isolation, in which establishing a reliable love relationship is fundamental. For Marcos, this task becomes considerably more difficult in a culture that discriminates against gay sexuality. The potential resolution of this conflict will require continuing personal transformations (cf. Chapin 2008). A more harmonious psychological and social integration of different aspects of his self (selves) and of his experiences of the world of the *misterios* and of human beings requires, and will continue to require, “a lot of work,” as Marcos describes his vocation. His religious experiences are ambiguous events that will require life‐long cognitive accommodation and assimilation (Luhrmann et al. 2010) and belie a facile characterization of his SP as either “central” and controlled versus “peripheral” and uncontrolled (cf. Lewis 1989). While psychological findings help explain why Marcos may have experienced more gaps of self‐control and identity than his contemporaries, anthropological constructs shed light on the practice and meanings that undergird his (and others') understanding of the sets of behaviors and experiences labeled SP.

In sum, SP follows different interpretation and valences throughout the lifespan, depending on developmental stages; relationships with others; the interaction of traits, states, and ritual practices; cultural (re)interpretations; and so on. We believe that our analysis integrating anthropological and psychological perspectives provide a more balanced and comprehensive view of SP than either discipline could provide singly, although of course it does not exhaust the ways in which this challenging experience can be meaningfully analyzed.
